# The potential role of dynamic thermal analysis in breast cancer detection

**DOI:** 10.1186/1477-7800-3-8

**Published:** 2006-04-03

**Authors:** M Salhab, LG Keith, M Laguens, W Reeves, K Mokbel

**Affiliations:** 1 St. George's Hospital, London, SW17 0QT, UK; 2 Northwestern University Medical School, Chicago, Illinois, USA; 3 Women's Medical Diagnostic Center. La Plata, Argentina; 4 Lifeline biotechnologies, Florida, USA

## Abstract

**Background:**

It is presently well accepted that the breast exhibits a circadian rhythm reflective of its physiology. There is increasing evidence that rhythms associated with malignant cells proliferation are largely non-circadian. Cancer development appears to generate its own thermal signatures and the complexity of these signatures may be a reflection of its degree of development. The limitations of mammography as a screening modality especially in young women with dense breasts necessitated the development of novel and more effective screening strategies with a high sensitivity and specificity. The aim of this prospective study was to evaluate the feasibility of dynamic thermal analysis (DTA) as a potential breast cancer screening tool.

**Methods:**

173 women undergoing mammography as part of clinical assessment of their breast symptoms were recruited prior to having a biopsy. Thermal data from the breast surface were collected every five minutes for a period of 48 hours using eight thermal sensors placed on each breast surface [First Warning System (FWS), Lifeline Biotechnologies, Florida, USA]. Thermal data were recorded by microprocessors during the test period and analysed using specially developed statistical software. Temperature points from each contra-lateral sensor are plotted against each other to form a thermal motion picture of a lesion's physiological activity. DTA interpretations [positive (abnormal thermal signature) and negative (normal thermal signature)] were compared with mammography and final histology findings.

**Results:**

118 (68%) of participating patients, were found to have breast cancer on final histology. Mammography was diagnostic of malignancy (M5) in 55 (47%), indeterminate (M3, M4) in 54 (46%) and normal/benign (M1, M2) in 9 (8%) patients. DTA data was available on 160 (92.5%) participants. Using our initial algorithm, DTA was interpreted as positive in 113 patients and negative in 47 patients. Abnormal thermal signatures were found in 76 (72%) out of 105 breast cancer patients and 37 of the 55 benign cases. Then we developed a new algorithm using multiple-layer perception and SoftMax output artificial neural networks (ANN) on a subgroup (n = 38) of recorded files. The sensitivity improved to 76% (16/21) and false positives decreased to 26% (7/27)

**Conclusion:**

DTA of the breast is a feasible, non invasive approach that seems to be sensitive for the detection of breast cancer. However, the test has a limited specificity that can be improved further using ANN. Prospective multi-centre trials are required to validate this promising modality as an adjunct to screening mammography especially in young women with dense breasts.

## Introduction

Breast cancer continues to be the most common malignancy in women. Epidemiological studies estimate that one in eight women will develop breast cancer during their lifetimes [1-4]. Moreover, one in five women with breast cancer will die of the disease despite the considerable advances in treatment. Given these circumstances, early detection of breast cancer is considered an important prognostic factor, and Reidy has aptly suggested that death from malignancy rather than its detection should be the point of reference in evaluating any screening programme [5].

Currently, mammography is considered the gold standard as a screening tool for the early detection of breast cancer. Unfortunately, it is a standard that does not always shine brightly in that wide variations exist in its sensitivity and specificity in published reports, as evidenced by a recent review by Sobti et al [6]. Moreover, it's limitations in young and premenopausal women with dense breast tissue strengthen the need to develop new modalities for the early detection of breast cancer, especially in this group of vulnerable patients. To this end, magnetic resonance imaging (MRI) has been shown to be more sensitive in the early detection of occult breast cancers, particularly in pre-menopausal women for whom the sensitivity of mammography is compromised [7] but with less specificity [8]. Additional modalities are still under development such as electrical impedance scanning (EIS) [9], mammary ductoscopy (MD) and proteomics of nipple aspirate fluid (NAF) and serum [10,11].

The establishment and growth of most tumours depend on the successful recruitment of new blood vessels into and around the tumour cells. This latter process, also known as angiogenesis, is dependent on the production of angiogenic growth factors by the tumour cells [12]. Because these new vessels lack smooth muscle fibers rendering them unreceptive to control by epinephrine [13,14], a more constant blood flow to the area increases the local temperature in the area surrounding the tumor.

It is now recognised that the breast exhibits a circadian rhythm that is reflective of its physiology [1,14]. The relationship between breast skin temperature and breast cancer was thoroughly examined by Gros et al [15,16]. The investigators found that the differences between the characteristics of rhythmic changes in skin temperature of clinically healthy and cancerous breasts were real and measurable.

The superficial thermal patterns measured on the surface of the breast are most likely related to tissue metabolism and vascularization within the underlying tissue. Such thermal patterns change significantly as a result of normal phenomena including the menstrual cycle, pregnancy and, more importantly, the pathologic process itself. Additionally, It is generally stated that cancer development, in most instances, represents the summation of large number of mutations that occur over years, each with its own particular histologic phenotype that can be seen in pre-menopausal mastectomy specimens [17-21]. Cancer development appears to generate its own thermal signatures, and the complexity or lack thereof may be a reflection of its degree of development [22-26].

Based on the understanding of the above pathological observations and the recent technological advances that have facilitated the recording of circadian rhythm variations of the breast[14] we prospectively aimed to examine the feasibility of a new potential screening method called the dynamic thermal analysis (DTA) for breast cancer detection.

## Methods and materials

Women referred to a private mammography unit in La Plata, Argentina as part of their clinical assessment for breast symptoms were recruited prior to having an indicated or otherwise planned biopsy. Institutional guidelines, including ethical approval and informed consent, were followed.

**Figure 1:**
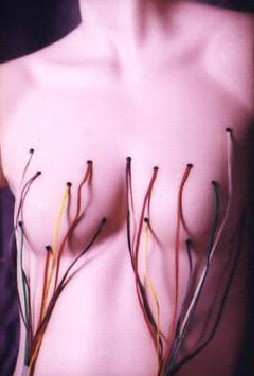
Thermal sensors of FWS applied to both breasts.

**Figure 2:**
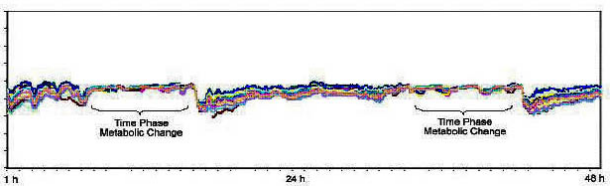
DTA in a patient with T1 breast cancer.

**Figure 3:**
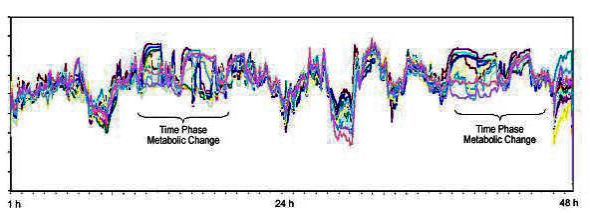
DTA in a patient with a fibroadenoma.

Thermal data from the breast surface were collected every five minutes for a period of 48 hours using eight thermal sensors placed on each breast surface [First Warning System (FWS), Lifeline Biotechnologies, Florida, USA]. Sensors are placed in anatomically critical positions elicited by data obtained from clinical examination and mammography as to where suspected cancers are located (Figure 1). Thermal data were recorded by microprocessors during the test period. More than 500 readings were collected in each patient. These data were analysed using specially developed statistical software called artificial neural networks (ANN). Temperature points from each contra-lateral sensor were plotted against each other to form a thermal motion pattern of a lesion's physiological activity (Figure 2, 3)

DTA interpretations [positive (abnormal thermal signature) and negative (normal thermal signature)] were compared with mammography and final histology findings.

## Results

A total of 173 women underwent mammography; their median age was 56 years (range: 17–85 Years). Fifty-eight women had a family history of breast cancer: 98 patients (56.6%) had a breast lump palpable of which 38 patients had a family history of breast cancer (38.7%)One hundred and eighteen (68%) of the 173 participating patients were found to have breast cancer on final histology with a tumour size range of 4–60 mm.

Mammography was diagnostic of malignancy (M5) in 55 patients (47%), indeterminate (M3, M4) in 54 patients (46%) and normal or benign (M1, M2) in 9 (8%) patients. (Table 1). Thermal data obtained from the breast surface were analysed in 160 participants (92.5%) using an initial algorithm. These data were interpreted as positive in113 patients and negative in 47 patients. Abnormal thermal signatures were found in 76 (72%) out of 105 breast cancer patients and 37 of the 55 benign cases (67%) (Specificity 32%) (Table 2)

**Table 1:** Mammography findings

**TEST**	**DISEASE**					
	
	**Cancer Present**	118		**Cancer Absent**	55	
Mammogram	True Positive	55	47%	True Negative	32	58%
	False Negative	9	8%	False Positive	7	13%
	*Indeterminate*	54	46%	*Indeterminate*	16	29%

**Table 2:** Results of the initial DTA

**TEST**	Cancer Patients			Cancer absent		
DTA	True Positive	**76**	72%	True Negative	18	33%
	False Negative	29	28%	False Positive	**37**	67%

We then developed a new algorithm using the multi-layer perception (MLP) with soft max output artificial neural networks (ANN) for use on a subgroup of participants (n = 38). MLP correctly detected cancer in 16 patients out of 21 participants improving the sensitivity to 76%. Furthermore, false positive cases were decreased to 26% (only 7 patients out of 27) increasing the specificity to 74%.

## Discussion

Currently mammography is the best available approach for the early detection of breast cancer in the general population with a sensitivity of 75–90% [2]. However, the positive predictive value is only 25% [3,4]. It is interesting to mention that 25–30% of breast cancers are found in pre-menopausal women. DTA is a safe and non-invasive technique that offers the advantage of providing information on the physiology of the breast, specifically the superficial thermal patterns that can be measured on the surface and relate to tissue metabolism and vascularization within the underlying tissue.

In healthy breasts, heat conductivity is constant in most cases and generally can be characterized in terms of circadian rhythm periodicity [27]. In contrast, the rhythms associated with malignant cells proliferation are largely non circadian and suggest that a circadian to ultradian shift may be a general correlation to neoplasia [14]. Due to the increased blood flow and the lack of receptivity in the newly formed vessels in malignancy, temperature production exhibits circadian rhythmic variations to a far lesser degree than is evident in the healthy breasts [13]. Our study demonstrates that 72% of patients with breast cancer have non circadian changes of the breast thermal patterns. However, the initial specificity of the test was very low. We then used the artificial neural network (ANN) to analyse the thermal data recorded in order to improve the sensitivity and specificity. In this type of analysis, the network learns by itself from a set of example solutions. It could also be divided into a supervised and unsupervised learning phase. The modification of the initial algorithm into a multi-layer perception (MLP) with soft max output artificial neural networks resulted in an increased sensitivity and specificity of the test. This new algorithm needs to be evaluated further in prospective multi-centre trials to validate these promising observations.

Appreciation of the clinical usefulness of heat-sensing devices in breast cancer has suffered because of poorly conceived attempts to exploit their diagnostic potential. Little attention has been paid to the thermal signal being an expression of immensely complicated biologic functions. The issue of false positives is still unclear; it may reflect an abnormality that can not be detected by conventional methods. The presence of non circadian rhythm in the absence of mammographic or clinical evidence of cancer does not preclude the presence of cancer in its very early stages. Patients with such signs might be considered high risk for breast cancer which may become clinically evident at a later date. This is supported by a previous French study investigating the long term follow up of individuals who had abnormal thermal signs in the absence of physical or mammographic findings [15]. However, further work is needed to evaluate this hypothesis.

The current DTA study has some other limitations. The ANN requires 540 input data for reliable analysis. Thermal data require a long time to be recorded (48 hours), and this may be considered as a downside of this technology. We are in the process of developing a new monitor which records thermal data every 5 seconds thus reducing the test time significantly.

If future validation studies confirm the reliability of DTA, then it can be used as an adjunct to mammography, especially in pre-menopausal women with dense breasts.
